# Significant association between genes encoding virulence factors with antibiotic resistance and phylogenetic groups in community acquired uropathogenic *Escherichia coli* isolates

**DOI:** 10.1186/s12866-020-01933-1

**Published:** 2020-08-05

**Authors:** Zahra Yazdanpour, Omid Tadjrobehkar, Motahareh Shahkhah

**Affiliations:** 1 Microbiology and Parasitology Department, Medical Faculty, Zabol University of Medical Sciences, Zabol, Iran; 2 Bacteriology and Virology Department, Medical Faculty, Kerman University of Medical Sciences, Kerman, Iran; 3 Microbiology Department, Medical Faculty, Zahedan University of Medical Sciences, Zahedan, Iran

**Keywords:** UPEC, Virulence associated genes, Antibiotic resistance, Phylogenetic groups

## Abstract

**Background:**

Antibiotic resistance is an increasing phenomenon in many bacterial pathogens including uropathogenic *Escherichia coli*. Hypothetical anti-virulent agents could be a solution, but first clear virulence associated gene-pool of antibiotic resistant isolates have to be determined. The aim of this study is to investigate the significant associations between genes encoding VFs with antibiotic resistance and phylogenetic groups in UPEC isolates.

**Results:**

The majority of 248 UPEC isolates belonged to phylogenetic group B2 (67.3%). The maximum and minimum resistance was attributed to amoxicillin (90.3%) and both fosfomycin and imipenem (1.6%) respectively. 11.3% of isolates were resistant to all antibiotic agents except that of imipenem, nitrofurantoin and fosfomycin. These highly resistant isolates were placed only in group B2 and D. The most prevalent virulence gene was *ompA* (93.5%). The *hlyA* was the only virulence gene that was significantly more prevalent in the highly resistant isolates. The *ompA*, *malX* and *hlyA* genes were obviously more abundant in the antibiotic resistant isolates in comparison to susceptible isolates. The *papC* gene was associated with amoxicillin resistance (*p*-value = 0.006, odds ratio: 26.00).

**Conclusions:**

Increased resistance to first line drugs prescribed for UTIs were detected in CA-UPEC isolates in our study.. Minimal resistance was observed against nitrofurantoin, fosfomycin and imipenem. Therefore, they are introduced for application in empirical therapy of UTIs. Fosfomycin may be the most effective antibiotic agent against highly resistant UPEC isolates. The presence of the *ompA*, *malX* and *hlyA* genes were significantly associated with resistance to different antibiotic agents. We assume that the ability of UPEC isolates to upgrade their antibiotic resistance capacity may occurs in compliance with the preliminary existence of specific virulence associated genes. But, more investigation with higher number of bacterial isolates, further virulence associated genes and comparison of gene pools from CA-UPEC isolates with HA-UPEC are proposed to confirm these finding and discovering new aspects of this association.

## Background

Urinary tract infection (UTI) is a widespread medical condition that involves both men and women and can occur frequently in different ages [1]. About 150 million new case of UTI worldwide are reported every year that most of them are women [2]. Many bacterial pathogens are known as the causative agents of UTI, but uropathogenic *Escherichia coli* (UPEC) is accounted as the most common agent (80–90%) of UTIs [2–4]. UPEC isolates are divided into the four main phylogenetic groups (A, B1, B2, and D) based on the some genomic sequences [5]. The most virulent UPEC strains are categorized in B2 and to a lesser extent in group D2. The A and B1 groups are less virulent and usually originate from fecal samples instead [6].

Establishment of UTI by UPEC is a multistep process that starts from overcoming of epithelial immune system and successful colonization of UPEC in the urinary epithelium. This process could be continue to the host tissue injury and even outspreading of bacteria to the blood circulation. Many virulence-factor associated genes are responsible for the production of virulence factors that are involved in each step. Structural factors such as outer membrane proteins, fimbriae and flagella are involved in the colonization process. The OmpA is a bacterial outer membrane protein that is involved in hole infection process including; adhesion, invasion, intracellular survival and immune system evasion of UPEC as well as many other pathogenic bacteria [7]. OmpA promotes some critical steps such as initial binding to bladder epithelium, chronic persistence and post invasion pathogenesis during UPEC infection [8]. Several types of surface exposed apparatus were detected in UPEC that some of the most known of them are type I fimbriae, P fimbriae and S fimbriae. Adhesions such as type 1 fimbriae of UPEC are encoded by the *fim* operon. Fim proteins have critical roles in assembly, folding and stabilizing of type I fimbriae. FimC chaperon is characterized to support correct folding of structural subunits to protrude from cell membrane, stabilizing them in periplasm and preventing them from unpredicted interactions [9]. The P fimbriae has heteropolymeric structure that is encoded by pap operon. This operon is part of a pathogenicity island that contains variable genes including *pap*A-K [2]. The PapC protein on the *E. coli* outer membrane is thought to be associated in outgoing translocation of pilin subunits through outer membrane and surface assembly [10]. Attachment of UPEC to renal epithelium via P fimbriae induces local inflammation, which subsequently leads to pain in urinary system [11]. The S fimbriae encoded by *sfa* genes which are highly homologous with *foc* operon that contains coding sequences of the Fic fimbriae proteins and these coding sequences could be exchanged between these two gene clusters. Therefore, the same obligatory genes like *focC* and *focD* exist in both gene clusters [12].

Agents responsible in iron-acquisition system and secreted toxins of UPEC are mainly enrolled in intracellular survival, immune system evasion and host tissue damage process [2]. In UPEC strains, iron is obtained from surrounding environment by production of various sidrophores, like aerobactin, salmochelin and yersiniabactin which are encoded by *iuc, iro* and *irp* gene clusters respectively [13, 14]. HlyA (α-haemolysin) is a secreted lipoprotein toxin that is able to disrupt host nucleated cells by pore forming activity. In addition, it can induce apoptosis in urinary tract epithelium and immune system associated cells [11, 15]. There are many Pathogenicity-associated islands (PAIs) accumulated in extra-intestinal *E. coli* strains and they carry different virulence associated genes, such as P fimbriae, Type I fimbriae, hemolysin, iron acquisition proteins, some bacteriocins and *malX* gene. The *malX* gene is part of a PAI that is associated with UPEC and it is known as phosphotransferase system enzyme II that uses glucose and maltose as the main substrates [16, 17].

Antibiotic resistance is an expanding phenomenon in many bacterial pathogens including UPEC [18]. Now today antibiotic resistance is a world spread problem and uncontrolled use of antibiotic agents can increase the antibiotic resistance in many countries including Iran. Therefore successful prescription of antibiotic agents will be limited day by day. Use of other alternatives such as presumptive anti-virulence agents could be a solution, but it will be operational if precise gene-pool that encodes virulence factors in resistant isolate and any association between these virulence associated genes and antibiotic resistance be investigated. In the present study we tried to investigate the antibiotic resistance patterns of community acquired UPEC isolates as well as the association between phylogenetic origin and virulence associated genes with antibiotic resistance in these isolates.

## Results

### Antibiotic resistance of different phylogenetic groups

All *E. coli* isolates were categorized into the four phylogenetic groups as follows: 167(67.3%) Group B2, 53(21.4%) Group D, 16(6.5%) Group A and 12(4.8%) Group B1.

Data obtained from disk diffusion test showed various frequency of antibiotic resistance including, 90.3% amoxicillin, 67.7% trimethoprim-sulfamethoxazole, 61.3% cephalexin, 59.7% ceftriaxone, 58.1% cefotaxime, 43.5% ciprofloxacin, 41.9% azithromycin, 40.3% ceftazidime, 27.4% gentamycin and minimum antibiotic resistance was detected against fosfomycin (1.6%), nitrofurantoin (3.2%) and imipenem (1.6%).

Totally 28(11.3%) out of 248 isolates were highly resistant. Resistance against amoxicillin, trimethoprim-sulfamethoxazole, cephalexin, ceftriaxone, cefotaxime, ciprofloxacin, azithromycin, ceftazidime and gentamycin were detected in these isolates. Twelve (42.9%) and 4 (14.3%) of these highly resistant isolates were resistant to nitrofurantoin and imipenem respectively, but resistance against fosfomycin was not observed in them.

Various resistance patterns were detected in four phylogenetic groups. Resistance to fosfomycin and imipenem were not identified in groups A and B1. All fosfomycin resistant isolates were placed in group B2. Resistance to gentamycin was not observed in group A. All group A and group B1 isolates were resistant to amoxycillin.

Logistic regression analysis showed no significant association between resistance or susceptibility to antibiotic agents with phylogenetic origins of UPEC isolates, except that of azithromycin. The Group B2 (*p*-value = 0.004, odds ratio: 0.195) and D (*p*-value = 0.003, odds ratio: 0.160) origins were associated with azithromycin susceptibility.

The bulk of the highly resistant isolates (82.1%) were placed in group B2 and they were absent from B1 and A groups (*p*-value =0.05, the Chi-square analysis), (detailed data not shown).

### Association of UPEC virulence genes with antibiotic resistance profile

The most prevalent virulence gene was *ompA* gene that was detected in 232(93.5%) of UPEC isolates. Prevalence of other six virulence genes were as follows: *fimC* 124(50%), *irp2* 156 (62.9%), *malX* 184(74.2%), *papC* 204(82.3%), *sfa/focCD* 52(21%) and *hlyA* 76(30.6%).

All seven virulence genes were found in four phylogenetic groups. The carriage of *papC* and *sfa/foc*CD were significantly associated with group B2 (Tables 1 and 2).

**Table 1:** Prevalence of seven virulence genes in various phylogenetic groups

Gene	Phylogenetic group n (%)			
	A (*n* = 16)	B1 (*n* = 12)	B2 (*n* = 167)	D (*n* = 53)
*ompA*	16 (100)	**8 (66.7)**	156 (93.4)	52 (98.1)
*fimC*	10 (62.5)	6 (50)	86 (41.5)	22 (41.5)
*irp2*	**12 (75)**	8 (66.7)	108 (64.7)	28 (52.8)
*malX*	9 (56.3)	7 (58.3)	127 (76)	41 (77.4)
*papC*	9 (56.3)	7 (58.3)	**144 (86.2)**	44 (83)
*sfa/foc*	3 (18.8)	1 (8.3)	**42 (25.1)**	**6 (11.3)**
*hly*	6 (37.5)	2 (16.7)	50 (29.9)	18 (34)

Bold n (%): Binary logistic regression analysis showed significant association (*p* ≤ 0.05)

**Table 2:** Binary logistic regression analysis of virulence-associated genes as predictor of phylogenetic origins

	Phylogenetic origin							
	B2		D		B1		A	
	OR	*P*	OR	*P*	OR	*P*	OR	*P*
*ompA*	0.858	0.796	5.149	0.117	**0.111**	**0.002**	2.092	0.998
*fimC*	1.452	0.191	0.591	0.104	0.610	0.454	1.271	0.682
*irp2*	0.933	0.823	0.697	0.269	0.667	0.630	**4.585**	**0.022**
*malX*	1.079	0.830	1.255	0.547	0.679	0.613	0.566	0.385
*papC*	**2.172**	**0.024**	1.078	0.870	0.298	0.052	0.065	0.000
*Sfa*	**2.366**	**0.025**	**0.380**	**0.038**	0.413	0.424	0.966	0.962
*hlyA*	0.696	0.256	0.950	0.892	1.208	0.834	3.860	0.064

*OR* odds ratio, *p p*-value; Bold *p*: Significant association (*p* ≤ 0.05) was shown by binary logistic regression analysis; When *p* ≤ 0.05, then OR > 1 means that virulence associated gene is introduced as predictor of that phylogenetic origin, but OR < 1 means that virulence associated gene is a predictor of other phylogenetic origins

Phylogenetic group A isolates showed reduced carriage of *papC* in comparison to the other groups. Instead, carriage of *irp2* was significantly associated with this group (Tables 1 and 2).

Prevalence of the *ompA* gene in group B1 was significantly less than the other groups (Tables 1 and 2).

The binary logistic regression analysis showed that *hlyA* was the only virulence gene that was significantly more prevalent in the highly resistant isolates showing resistance to all antibiotic agents except that of imipenem, nitrofurantoin and fosfomycin (*p*-value = 0.000, odds ratio: 8.000), (detailed data not shown).

Prevalence of different virulence genes in antibiotic resistant isolates were studied separately for each antibiotic agents. Among 9 investigated virulence genes, *sfa/focCD*, *irp2*, and *fimC* were absent in isolates that were resistant to imipenem. We did not observe *sfa/focCD* gene in nitrofurantoin resistant isolates and *hlyA*, *papC*, *malX*, *irp2* and *fimC* were absent from fosfomycin resistant isolates (Table 3).

**Table 3:** Frequency of different virulence genes in antibiotic resistant isolates in comparison to susceptible isolates

Antibiotic agents	R or S (n)	Virulence genes n (%)						
		***hlyA***	***sfa/foc***	***papC***	***malX***	***Irp2***	***fimC***	***ompA***
Azithromycin	**R**(140)	52 (37.1)	24 (17.1)	116 (82.9)	104 (74.3)	92 (65.7)	68 (48.6)	136 (97.1)
	**S**(108)	24 (22.2)	28 (25.9)	88 (81.5)	80 (74.1)	64 (59.3)	56 (51.9)	96 (88.9)
Amoxicillin	**R**(228)	72 (31.6)	44 (19.3)	192 (84.2)	172 (75.4)	136 (69.6)	112 (49.1)	216 (94.7)
	**S**(20)	4 (20)	8 (40)	12 (60)	12 (60)	20 (100)	12 (60)	16 (80)
Imipenem	**R**(8)	8 (100)	0 (0)	8 (100)	8 (100)	0 (0)	0 (0)	8 (100)
	**S**(240)	68 (28.3)	52 (21.7)	196 (81.7)	176 (73.3)	156 (65)	124 (51.7)	228 (93.3)
Gentamycin	**R**(68)	28 (41.2)	4 (5.9)	60 (88.2)	60 (88.2)	40 (58.8)	40 (58.8)	60 (88.2)
	**S**(180)	48 (26.7)	48 (26.7)	144 (80)	124 (68.9)	116 (64.4)	84 (46.7)	172 (95.6)
Ciprofloxacin	**R**(116)	48 (41.4)	24 (20.7)	92 (79.3)	100 (86.2)	68 (58.6)	48 (41.4)	112 (96.6)
	**S**(132)	28 (21.2)	28 (21.2)	112 (84.8)	84 (63.6)	88 (66.7)	76 (57.6)	120 (90.9)
Cefotaxime	**R**(164)	56 (34.1)	32 (19.5)	136 (82.9)	128 (78)	100 (61)	72 (43.9)	156 (95.1)
	**S**(84)	20 (23.8)	20 (23.8)	68 (81)	56 (66.7)	56 (66.7)	52 (61.9)	76 (90.5)
Fosfomycin	**R**(4)	0 (0)	4 (100)	0 (0)	0 (0)	0 (0)	0 (0)	4 (100)
	**S**(244)	76 (31.1)	48 (19.7)	204 (83.6)	184 (75.4)	156 (63.9)	124 (50.8)	228 (93.4)
Cephalexin	**R**(184)	68 (37)	40 (21.7)	156 (84.8)	140 (76.1)	108 (58.7)	80 (43.5)	180 (97.8)
	**S**(64)	8 (12.5)	12 (18.8)	48 (75)	44 (68.8)	48 (75)	44 (68.8)	52 (81.3)
Ceftazidime	**R**(112)	40 (35.7)	20 (17.9)	92 (82.1)	92 (82.1)	64 (57.1)	48 (42.9)	108 (96.4)
	**S**(136)	36 (26.5)	32 (23.5)	112 (82.4)	92 (67.6)	92 (67.6)	76 (55.9)	124 (91.2)
Ceftriaxone	**R**(160)	56 (35)	32 (20)	136 (85)	128 (80)	100 (62.5)	68 (42.5)	152 (95)
	**S**(88)	20 (22.7)	20 (22.7)	68 (77.3)	56 (63.6)	56 (63.6)	56 (63.6)	80 (90.9)
Nitrofurantoin	**R**(24)	16 (66.7)	0 (0)	16 (66.7)	24 (100)	8 (33.3)	8 (33.3)	24 (100)
	**S**(224)	60 (26.8)	188 (83.3)	160 (71.4)	148 (66.1)	148 (51.8)	116 (51.8)	208 (92.9)
Cotrimoxazole	**R**(176)	52 (29.5)	32 (18.2)	144 (81.8)	128 (72.7)	108 (61.4)	84 (47.7)	164 (93.2)
	**S**(72)	24 (33.3)	20 (27.8)	60 (83.3)	56 (77.8)	48 (66.7)	40 (55.6)	68 (94.4)

*R* Resistant, *S* Sensitive

The *fimC*, *irp2* and *sfa/focCD* genes were significantly more prevalent in isolates sensitive to different class of antibiotic agents in comparison to resistant isolates. On the contrary, *ompA*, *malX* and *hlyA* genes were obviously more abundant in the isolates resistant to different antibiotic agents (Table 4). The *papC* gene was associated with amoxicillin resistance (*p*-value = 0.006, odds ratio: 26.00). Prevalence of different virulence genes among resistant isolates is presented in Table 3.

**Table 4:** Binary logistic regression analysis of virulence-associated genes as predictor of resistance to various antibiotic agents

	Resistance to antibiotic agents																							
	Azithromycin		Amoxicillin		Imipenem		Gentamycin		Ciprofloxacin		Cefotaxime		Fosfomycin		Cephalexin		Ceftazidime		Ceftriaxone		Nitrofurantoin		Cotrimoxazole	
	OR	*p*	OR	*p*	OR	*p*	OR	*p*	OR	*p*	OR	*p*	OR	*p*	OR	*p*	OR	*p*	OR	*p*	OR	*p*	OR	*p*
*ompA*	**4.919**	**0.010**	3.386	0.151	0.000	0.999	0.493	0.201	2.012	0.253	2.010	0.197	0.872	1.000	**6.493**	**0.002**	2.791	0.092	1.612	0.392	2.406	1.000	0.816	0.745
*fimC*	0.313	0.753	1.504	0.498	0.000	0.995	1.726	0.080	**0.563**	**0.039**	**0.482**	**0.008**	0.233	1.000	**0.377**	**0.003**	0.616	0.074	**0.457**	**0.005**	**0.342**	**0.045**	0.648	0.136
*irp2*	1.618	0.850	0.000	0.995	0.000	0.995	0.672	0.240	1.348	0.363	1.052	0.862	0.456	1.000	0.724	0.360	0.839	0.559	1.254	0.455	1.111	0.857	0.876	0.668
*malX*	0.939	0.855	1.842	0.457	0.000	0.999	**4.700**	**0.000**	**5.170**	**0.000**	1.609	0.118	0.000	0.994	0.878	0.736	**2.235**	**0.011**	**2.058**	**0.017**	3.121	0.996	0.764	0.425
*papC*	0.301	0.673	**26.00**	**0.000**	2.344	1.000	1.427	0.483	**0.189**	**0.000**	0.756	0.486	0.027	1.000	1.496	0.344	0.610	0.204	0.970	0.942	0.000	0.995	1.164	0.732
*Sfa*	**0.514**	**0.041**	**0.115**	**0.001**	0.79	1.000	**0.151**	**0.001**	0.697	0.287	0.584	0.115	3.208	0.993	0.752	0.478	0.523	0.053	0.600	0.137	0.000	0.997	0.578	0.094
*hlyA*	**1.887**	**0.033**	1.711	0.482	1.167	0.994	1.643	0.140	**2.651**	**0.002**	1.348	0.370	2.166	1.000	**3.433**	**0.003**	1.246	0.478	1.493	0.221	0.000	0.996	0.875	0.676

*OR* odds ratio, *p p*-value; Bold *p*: Significant association (*p* ≤ 0.05) was shown by binary logistic regression analysis; When *p* ≤ 0.05, then OR > 1 means that virulence associated gene is introduced as predictor of resistance to antibiotic agent, but OR < 1 means that virulence associated gene is a predictor of susceptibility to antibiotic agent

## Discussion

Antibiotic resistance is a globally increasing problem especially in developing countries. This is due to greater access to antibiotic drugs, over-prescription or arbitrary use of antibiotic agents.

The highest resistance were detected against amoxicillin and trimethoprim-sulfamethoxazole in our assessments. High rate of resistance to first line antibiotics that are prescribed to treat uncomplicated UTIs such as amoxicillin and trimethoprim-sulfamethoxazole were also reported in other studies from Iran and other countries [14, 18–21]. This condition is conducive to uncontrolled application of empirical therapy with fluoroquinolones, third generation cephalosporins and even new macrolides like azithromycin. Therefore, increased resistance to these antibiotic agents could be expected as well it is observable in our results.

Aminoglycosides like gentamycin and carbapenem antibiotics are not usually prescribed for outpatients UTIs in Iran in this regard, lower rate resistance against them is observed.

Evidence indicates that, higher antibiotic resistance rate is expected in hospital acquired bacteria in comparison to community acquired bacteria [22]. But antibiotic resistance increases continually in community acquired bacteria. Our results showed a high prevalence of antibiotic resistance against different antibiotic agents in CA-UPEC isolates. Thus, continuous screening of resistance patterns in CA-bacteria like CA-UPEC is mandatory.

Our finding revealed that, 11.3% of isolates were resistant to all antibiotic agents except that of imipenem, nitrofurantoin and fosfomycin. Therefore, nitrofurantoin, fosfomycin and carbapenem agents like imipenem are suitable candidates for empirical therapy of UTIs in outpatients. None of the highly resistant isolates were resistant to fosfomycin. Hence, fosfomycin is introduced as the most effective antibiotic agent against highly resistant CA-UPEC isolates.

UPEC outer membrane proteins (e.g., OmpA), are highly conserved and common among different strains [2, 8]. This is concomitant with our results indicating the highest prevalence of *ompA* gene among different virulence genes (Table 1). Statistical analysis showed that the prevalence of *ompA* was not significantly different between A, B2 and D phylogenetic groups. But, the prevalence of *ompA* was significantly lower in Group B1 (*p*-value = 0.003, odds ratio: 0.029).

The *papC* gene was the second common virulence factor and the most common adhesion among the UPEC isolates in the present study. In this regard, P fimbriae was reported as the second common virulence factor of UPEC Bien et al. [11]. More association of *papC* with UPEC pathotypes in comparison to other pathotypes has been also reported by Titilawo et al. [23]. High prevalence of P fimbriae associated genes in our study may be attributed to special colonization properties of CA-UPEC isolates that were obtained from this geographical area and show the importance of P fimbriae as the major adhesion in these isolates.

Phylogenetic origins analysis showed that, the most numbers of UPEC isolates belonged to Group B2 (67.3%) and subsequently Group D (21.4%) respectively. Similar results were obtained from other studies [24]. The most of the VFs were not significantly correlated with any of phylogenetic groups, except for *papC* and *sfa/foc*CD genes which were predominantly more prevalent in Group B2 (Tables 1 and 2). The significant association between the *papC* and *sfa/focCD* genes with Group B2 is reasonable, because most of the UPEC isolates are categorized in B2 phylogenetic group. Therefore, *papC* and *sfa/focCD* genes are introduced as a predictor of Group B2 origin.

The present study findings showed a strong association between the *irp2* gene and Group A origin (Table 2). The *irp2* gene of *Yersinia* is present in different genera of the family *Enterobacteriaceae* including *E. coli, Klebsiella*, *Citrobacter* and others [25]. On the other hand, *irp2* is considered as one of the virulence associated genes in diarrheagenic *E. coli* that is mainly allocated in phylogenetic Group A [26]. Therefore, the *irp2* gene is introduced as a predictor for Group A origin. Therefore, uropathogenic ability of some diarrheagenic *E. coli* strains may be considered as a hypothesis again in our study, as was reported previously [26].

Logistic regression analysis also showed that *papC* gene is a predictor for amoxicillin resistance (*p*-value = 0.006, odds ratio: 26.00). A parallel finding has been reported by Karami et al., in which they introduced *papC* gene as a predictor for ampicillin resistance [6].

**Fig. 1:**
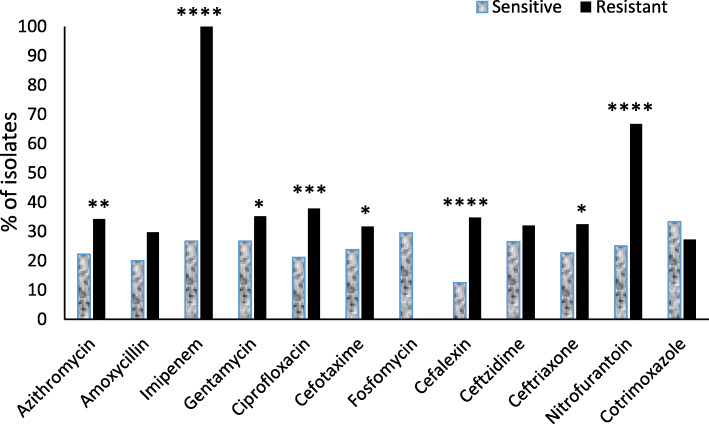
Prevalence of isolates which contained three virulence associated genes (*ompA* + *malX* + *hlyA*)*,* in sensitive and resistant isolates. Prevalence (%) of isolates which contained three virulence associated genes (*ompA* + *malX* + *hlyA*) in sensitive isolates (white columns) in comparison to resistant isolates (black columns) have shown separately for any of the tested antibiotic agents. *p < 0.2, **p < 0.05, ***p < 0.01 and ****p < 0.001 (Fisher’s exact test analysis)

Data obtained from logistic regression analysis showed reduced prevalence of some virulence genes such as *irp2*, *fimC* and *sfa/focCD* in antibiotic resistant isolates in comparison to antibiotic susceptible isolates. On the contrary, the *ompA*, *malX* and *hlyA* genes were obviously more abundant in the isolates resistant to different antibiotic agents (Table 4). Moreover, the isolates that contained all three genes (*ompA* + *malX* + *hlyA)* were significantly (confidence level ≥ 95%) more resistant to azithromycin, imipenem, ciprofloxacin, cephalexin, ceftriaxone, nitrofurantoin, and even with lower confidence level (80–90%) to cefotaxime and gentamycin in comparison to other isolates (Fig. 1). Therefore, the *ompA*, *malX* and *hlyA* genes are introduced as possible predictors of antibiotic resistance in CA-UPEC isolates.

The lower prevalence of genes encoding type 1 fimbriae, P fimbriae and also *hlyA* gene in quinolone-resistant isolates in comparison to susceptible isolates were reported in some investigations [27–29]. Our finding confirmed them about type1 fimbriae and genes encoding P fimbriae, but it’s in contrary with the results attributed to *hly* genes. The logistic regression analysis showed that, the prevalence of this gene was significantly more prevalent in ciprofloxacin resistant isolates in comparison to susceptible isolates (Tables 3 and 4). This contradiction is reasonable because, the gradual evolutionary mechanisms responsible for antibiotic resistance in more virulent isolate could be arises any time and independently from any changes in genes encoding VFs, just in consequence of the continuous encounter with antibiotic agents. This finding is in line with other studies reported previously [30–32].

## Conclusion

The UPEC isolates have very versatile gene pools contains some virulence associated genes that might be more intrinsic in comparison to antibiotic resistance genes. The results obtained from the present study showed that some surface exposed adhesions and iron-acquisition system are obviously associated with antibiotic susceptibility. On the contrary, *ompA* and some of the other pathogenicity-island related genes predominantly are associated with antibiotic resistance. We believe that antibiotic resistance acquisition may occur in compliance with the preliminary existence of specific genetic background including genes encoding VFs such as *ompA*, *malX* and *hlyA* in CA-UPEC isolates and descriptive reasons maybe revealed later. Although, our results showed an association between antibiotic resistance pattern and some virulence associated genes, but more investigation with higher number of bacterial isolates are needed to confirm these findings and the comparison of gene pools from CA-UPEC isolates with HA-UPEC could be more beneficial in this respect. Studies on further virulence associated genes and some resistance genes like ESBLs could be resulted in the discovery of new aspects of this association.

## Methods

### Bacterial isolates and culture conditions

All bacterial isolates were obtained from urine samples of symptomatic outpatients who referred to medical laboratories of Zabol, southeast of Iran, during 2018–2019. Patients with a history of antibiotic use in the recent month were excluded. Agar plates containing the harvested bacteria were transferred to the microbiology laboratory of medical faculty regularly. Bacterial isolates were inoculated primarily on EMB agar plates and incubated at 37 °C for 24 h. The grown colonies with metallic shine appearance were confirmed by biochemical tests. All isolates were inoculated into the 1.5 ml microtubes containing Muller-Hinton broth with 20% glycerol and preserved at − 70 °C for subsequent use.

### Antibiotic susceptibility assessment

Antibiotic susceptibility profiles were determined by standard disc diffusion test, Kirby-Bauer method, according to CLSI guidelines [33]. Susceptibility and resistance criteria were estimated based on CLSI M100-S27 protocol [34]. ATCC *E.coli* 25,922 was used as control strain in disk diffusion susceptibility test. Twelve antibiotic disk in different classes were used including β-lactames (amoxicillin 30 μg, cephalexin 30 μg, ceftriaxone 30 μg, cefotaxime 30 μg, ceftazidime 30 μg), aminoglycosides (gentamicin 10 μg), phosphonic antibiotics (fosfomycin 200 μg), nitrofuran antibiotics (nitrofurantoin 300 μg), carbapenems (imipenem 10 μg), sulfonamides (trimethoprim-sulfamethoxazole 25 μg), quinolones (ciprofloxacin 5 μg) and macrolides (azithromycin 15 μg), that all were from Rosco Diagnostica (Denmark), were used in susceptibility assessments.

### DNA extraction and PCR conditions

All UPEC isolates were cultured in Luria-Bertani broth (Merck, Germany) and incubated for 24 h at 37 °C. Genomic DNA was prepared from harvested bacteria by boiling lysis method. Briefly, bacterial suspensions in distilled water were boiled at 95 °C in water bath for 10 min. After centrifugation, the supernatant was stored at − 20 °C. Aliquots of 2.5 μl template DNA were used for PCR [24, 35]. The sequences that used as primer were acquired from two previous studies [5, 36]. All primers were purchased from (Bioneer, South Korea). Polymerase chain reactions were conducted by Biometra termocycler (T-Gradient thermoblock, Germany).

### Phylogenetic classification

Genomic DNA was extracted from all *E. coli* isolates and PCR amplification was performed with specific primers incorporated to *chuA* and *yjaA* genes and TspE4.C2 sequences. Each isolate was allocated into one of the four phylogenetic groups (A, B, B2 & D) based on the existence of the PCR products as described earlier by Clermount, et al. [5]. In summary, the phylogenetic groups were assigned according to the following genotypes: group B2 (*chuA*+/*yjaA*+), group D (*chuA*+/*yja*A−), group B1 (*chuA*−/*TspE4.C2*+) and group A (*chuA*−/*TspE4.C2*−).

### Statistical analysis

Data analysis was performed by SPSS software version 18. The Chi-square and Fisher’s exact test were used to compare the association of genes encoding VFs with different variables such as antibiotic resistance and phylogenetic groups. Binary logistic regression analysis was used to assess the role of virulence genes and phylogenetic origin as predictors of resistance to different antibiotic agents. The *p-*value ≤0.05 was considered as significant.

## Supplementary information

**Additional file 1.** Table of primers & PCR conditions

## Data Availability

The datasets used and/or analyzed during the current study are available from the corresponding author on reasonable request.

## Abbreviations

UTI: Urinary tract infection
UPEC: Uropathogenic *Escherichia coli*
VF: Virulence Factor
CLSI: Clinical and Laboratory Standards Institute
ATCC: American Type Culture Collection
EMB: Eosin Methylene Blue
PCR: Polymerase Chain Reaction
CA-UPEC: Community Acquired Uropathogenic *Escherichia coli*
HA-UPEC: Hospital Acquired Uropathogenic *Escherichia coli*
PAI: Pathogenicity Associated Islands
