# Supplementary material for: Significant association between genes encoding virulence factors with antibiotic resistance and phylogenetic groups in community acquired uropathogenic Escherichia coli isolates
Source: BMC Microbiol. 2020 Aug 5;20:241. doi: 10.1186/s12866-020-01933-1 (PMC7409443; doi:10.1186/s12866-020-01933-1)
Supplement: Supplementary file 1 — Additional file 1. Table of primers & PCR conditions [file 12866_2020_1933_MOESM1_ESM.docx]

| Genes or DNA fragment | Description | Primer sequences(5’→3’;  F: forward, R: reverse) | Source | PCR condition  (30 cycles) | | | |
| --- | --- | --- | --- | --- | --- | --- | --- |
|  |  |  |  | D | A | E | |
| *papC* | P fimbriae | F:TGATATCACGCAGTCAGTAGC  R: CCGGCCATATTCACATAAC | 23 | 30s at 94 ºC | 30s at 54 ºC | | 1m at 72 ºC |
| *hlyA* | Haemolysin A | F: GTCCATTGCCGATAAGTTT  R: AAGTAATTTTTGCCGTGTTTT | 23 | 30s at 94 ºC | 30s at 52 ºC | | 1m at 72 ºC |
| *malX* | PAI marker CFT073 | F:GACATCCTGTTACAGCGCGCA  R:CGCCACCAATCACAGCCGAAC | 23 | 30s at 94 ºC | 40s at 60 ºC | | 1m at 72 ºC |
| *ompA* | Outer membrane protein A | F: AGCTATCGCGATTGCAGTG  R: GTGTTGCCAGTAACCGG | 23 | 30s at 94 ºC | 30s at 52 ºC | | 1m at 72 ºC |
| *sfa/focCD* | S-fimbriae/ F1C fimbriae | F: CCTGACTCATCTGAAACTGCA  R: GGAGAACTGGGTGCATCTTA | 23 | 30s at 94 ºC | 40s at 58 ºC | | 1m at 72 ºC |
| *fimC* | Type 1 fimbriae | F: GGTAGAAAATGCCGATGGTG  R: CGTCATTTTGGGGGTAAGTGC | 23 | 30s at 94 ºC | 30s at 54 ºC | | 1m at 72 ºC |
| *irp2* | Iron acquisition protein | F: AAGGATTCGCTGTTACCGGAC  R: TCGTCGGGCAGCGTTTCTTCT | 23 | 30s at 94 ºC | 30s at 57 ºC | | 1m at 72 ºC |
| **chuA* | Heme receptor gene | F: GACGAACCAACGGTCAGGAT  R: TGCCGCCAGTACCAAAGACA | 5 | 30s at 94 ºC | 30s at 55 ºC | | 40s at 72 ºC |
| **yjaA* | Stress response protein | F: TGAAGTGTCAGGAGACGCTG  R: ATGGAGAATGCGTTCCTCAAC | 5 | 30s at 94 ºC | 30s at 55 ºC | | 40s at 72 ºC |
| ***TspE4C2 | Anonymous DNA fragment | F: GAGTAATGTCGGGGCATTCA  R: CGCGCCAACAAAGTATTACG | 5 | 30s at 94 ºC | 30s at 55 ºC | | 40s at 72 ºC |

Primers and conditions used for PCR amplification of virulence associated genes and phylogenetic allocation

* These are only used in phylogenetic allocation. D: denaturation step, A: annealing step, E: extension step. All cycles were started by an initiation step; 7 min at 94ºC and finished with a termination step 7 min at 72ºC.
